# The Past Informs the Present, Academic New Media Pitfalls: A Primer for Plastic Surgeons

**DOI:** 10.1097/GOX.0000000000002178

**Published:** 2019-04-01

**Authors:** Kristopher M. Day, Rod J. Rohrich, Alexander M. Spiess

**Affiliations:** From the * Department of Plastic Surgery University of Texas Medical Branch, Austin, Tex.; † Department of Plastic Surgery University of Texas Southwestern Medical Center Dallas, TX; ‡ Dallas Plastic Surgery Institute, Dallas, Tex.; § Department of Plastic Surgery University of Pittsburgh Medical Center Pittsburgh, Penn.;.

## Abstract

Mass communication has undergone a “new media” revolution, which includes the rise of digital, online, and social media. The impact of new media on academic processes, however, has been underappreciated. The rise of Web-based virtual platforms has profoundly impacted the way plastic surgeons publish, store, exchange, and analyze scholarly biomedical information. This new media academic phenomenon refers to electronic mechanisms with the capacity to supplant traditional publication methods, which typically rely on printed documents in the physical domain. Although such tools can be efficient and user-friendly, they also make users vulnerable to exploitation. Notable examples reveal a relative lack of regulation, oversight, reliable rating scales, user authentication, and ethical accountability in the virtual space. As with any new technique, education is key and knowledge is power. In this article, online resources related to healthcare and the practice of plastic surgery are reviewed and summarized, including open access, mega-indices, whitelists, and electronic alerts. New media provides powerful knowledge-sharing tools that can help execute scholarly endeavors, communicate between professionals, and educate the public. However, it is essential for plastic surgeons to appreciate the caveats of new media academic processes to avoid unscrupulous practices of those that may seek to manipulate these Web-based systems. This article outlines the key pitfalls associated with online information streams to better inform plastic surgeons how to navigate new media-based scholarly processes.

(New) media is one of the new surgical skills. If you don’t exist on it then you don’t exist according to the public…It is important to get involved to dilute dodgy practices by non-plastic surgeons…and we’re the ones who should be doing that.

—Olivier Branford^1^

## INTRODUCTION: CHANGE, THE ONLY CONSTANT

The *Diamond Sutra* initiated mass communication as the first “printed” text and included the description “for universal free distribution.”^2^ This seems to have predicted the evolution of mass media itself by nearly 3 millennia. “New media,” or digital, online, and social media platforms, have democratized information access, exchange, and distribution more than any development since the launch of the World Wide Web in 1991.^3,4^ Electronic resources now have the capability to supplant their physical counterparts, which has impacted industries like newspapers, books, music records, travel planning, investment brokerage, banking, and big box retailers.^5^ The transformation of postal mail, compact disks, video cassettes, or satellite television dishes to their digital counterparts are but a few obvious examples. As a profession with a high public profile that relies on visual results appealing to surgeons, patients, and the lay public alike, plastic surgery has been significantly affected by the new media revolution.

The influence of new media on marketing in plastic surgery is well described. The debate continues about the superiority of Facebook, Twitter, Instagram, SnapChat, Pinterest, or YouTube, but the common use of one platform or another is the one constant.^6^ An estimated 70% of adults now use the Internet as their first source for health information,^7^ including approximately 90% of plastic surgery patients.^8^ Web-based virtual platforms can facilitate knowledge sharing for public education, interspecialty and intraspecialty communication, and execution of scholarly endeavors. As previously tangible materials convert into their virtual clones, new logistical, ethical, security, and quality concerns arise. Online tools can enhance efficiency and accessibility, but they also increase users’ vulnerability to exploitation given a lack of regulation, oversight, rating scales, authentication protocols, and enforceable accountability. An American Society of Plastic Surgeons survey reports that few plastic surgeons suspect a negative impact from new media platforms but also believe that greater oversight is necessary.^9^

The impact of new media on academic processes in plastic surgery is less appreciated. Transforming the way plastic surgeons store, exchange, publish, and analyze of scholarly biomedical information has profound implications for evidence-based patient care. New media has ushered in strictly Web-based academic activities (Table 1). Online journals may now publish ahead of or in lieu of print, otherwise known as “eprinting.” An article’s supplementary online multimedia content might accrue more views than its printed source content. Digitalized “big data,” automated data management, and electronic medical records have all but eliminated traditional pencil-and-paper scientific rigor. Open access journals, publication mega-indices, and consolidated article email alerts have changed the way we access scientific content. Altmetrics, online reviewer credentialing, and Web-based manuscript marketing services have transformed the profile of the academic plastic surgeon.

**Table 1.**
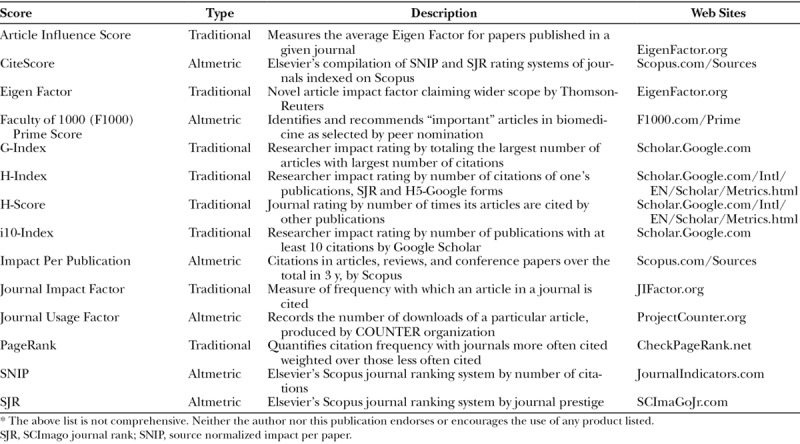
Academic Impact Scoring Systems

The cross talk between evidence-based medicine and new media is accelerating, raising the question whether the 2 are irrevocably conjoined. Oversight for new media academic processes lags behind the explosion of websites touting themselves as online knowledge depots. Fortunately, the most egregious faux pas in new media scholarship have occurred outside of the realm of plastic surgery, perhaps due to the shrewd stewardship of plastic surgery editors compared with other scientific fields overall. The field still remains susceptible, given that much of the plastic surgery literature is of relatively low level of evidence and possesses a popular appeal that may attract charlatans.^8–13^ As with any new tool, education is key and knowledge is power. This article outlines new media academic pitfalls to prepare plastic surgeons to best navigate these online scholarly processes. Through contrasting new media academic practices with their historic underpinnings, we see how far we have come and perhaps how far we still have to go.

## THE RISE OF ALTMETRICS

Jorge Hirsch devised a method to quantify an individual academician’s impact on the greater scientific community called the “H-Index,” a standard metric for university promotion.^14^ Associate professors in plastic surgery have H-indices of approximately 9, professors about 15, and Nobel laureate typically over 70.^15,16^ Similar to an author’s H-Index, the “H-Score” quantifies a journal’s rating by the number of times its articles are cited by other publications, and many other scoring systems exist (Table 1). These are examples of traditional scientific impact metrics, which tabulate the frequency of citations of one publication by others.^17^

The current conundrum is what to make of new media-based rating systems, which are “mention,” “like,” or other “engagement”-based instead of citation-based (Table 2).^18^ Altmetrics, “article-level,” or “alternative” scientific impact scores quantify the number of new media hits generated by a publication. Depending on one’s perspective, they are either more or perhaps much less reflective of an article’s import to science and humanity. Citations and altmetric scores are not always correlated.^18–20^ Therefore, journals achieve a different impact factor or ranking based on which system is employed (Table 3).

**Table 2.**
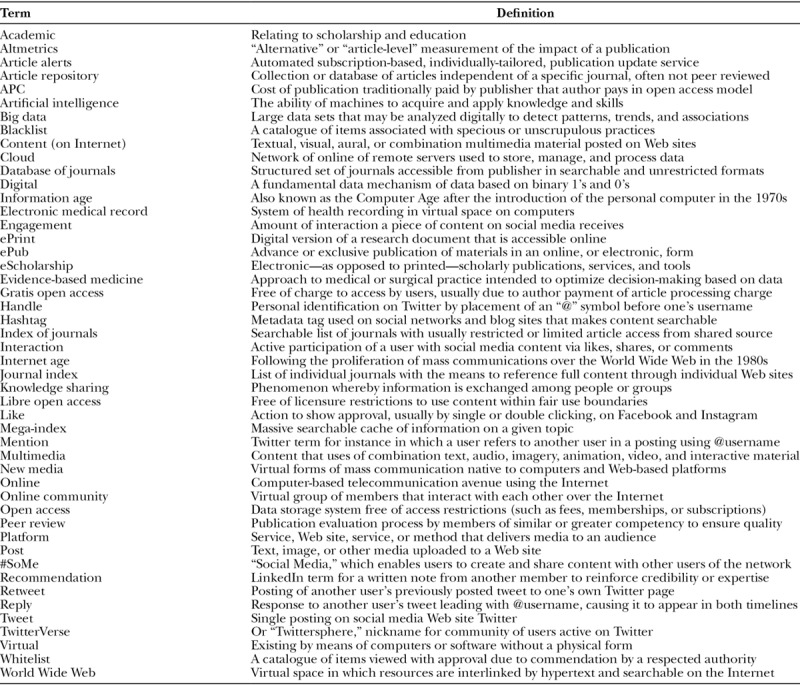
Academic New Media Terminology*

**Table 3.**
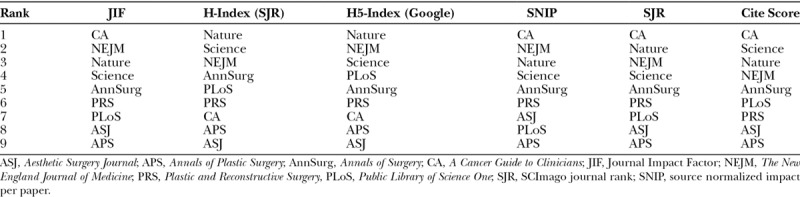
Prominent Journals’ Conventional and Altmetric Relative Rankings^21–24^

With over 150,000 Twitter followers and countless invited presentations on the topic, *Plastic and Reconstructive Surgery*’s Social Media Editor, Olivier Branford, is a thought leader on new media in plastic surgery. His report on the use of “#PlasticSurgery” on Twitter demonstrated that over 70% of posts using this hashtag were made by members of the lay public rather than plastic surgeons.^25^ The paper itself has generated the journal’s fourth highest online traffic rate of all time (altmetric score: 563). Compare this to Barack Obama, the only sitting president to publish a peer-reviewed scientific article, an update on the status of the United States’ healthcare system in 2016, whose altmetric score continues to climb into the 8,000s. Lay public account for over 70% of such interactions with 17% and 7% attributed to scientists and healthcare professionals, respectively.^26^

Contrasting altmetrics with the traditional citation recording system, we look at the most cited peer-reviewed publication in print, a 1,951 paper that describes a method for quantifying protein that has over 300,000 citations.^27^ National Academy of Sciences member Oliver H. Lowry’s report of “Protein Measurement with the Folin Phenol Reagent” has not been tweeted as often as Dr. Branford’s “Concepts in Aesthetic Breast Dimensions: Analysis of the Ideal Breast.” In fact, the eye-catching breast aesthetics paper has been engaged on new media over 5 times more often than in peer-reviewed publications.^28^ Although Dr. Lowry’s is the most cited paper of all time, it cannot be found on Altmetric.com.^29^

The debate for traditional versus progressive academic ratings systems could go on ad infinitum. Plastic surgeons need only realize that these rating systems have become popular adjunct metrics for academic significance. Given the field’s high public profile and apt fodder for new media content, the growth of altmetrics in academic plastic surgery has likely only begun (Table 4).

**Table 4.**
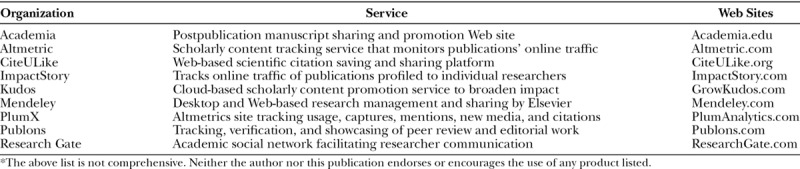
Altmetrics and Publication Promotion Web Sites

## OPEN ACCESS: PROS AND CONS

**Fig. 1.**
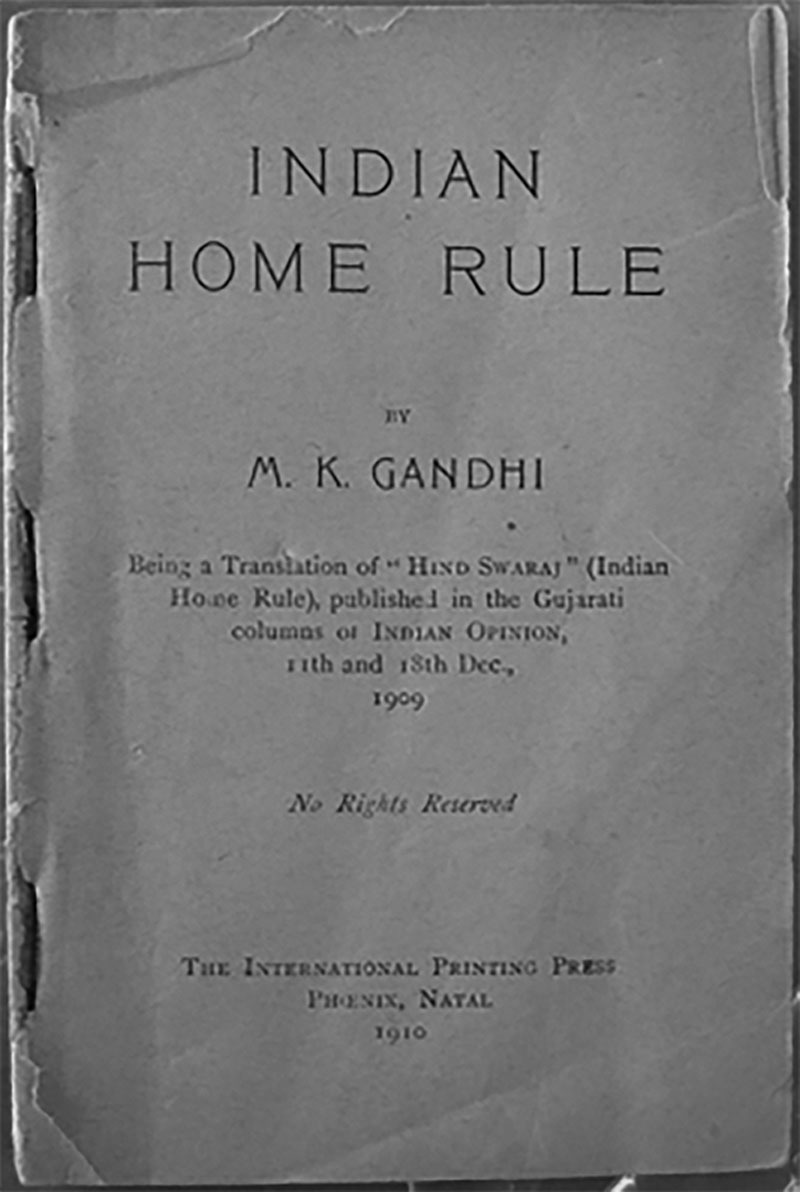
Gandhi’s^30^ *Indian Home Rule* english translation cover. The phrase “No Rights Reserved” was published on the cover of the English translation of *Indian Home Rule*. This was done to maximize the distribution of Gandhi’s influential text to optimize its political impact.

Mahatma Gandhi’s *Indian Home Rule* is credited with precipitating the end of British imperialism in India.^30^ It was also one of the first open access publications, printed with the phrase “No Rights Reserved” on the cover, making it illegal to restrict access to its text (Fig. 1). The scholarly publication market, by contrast, has traditionally been limited to fee-based access with significant economic implications. A single international publisher can generate over $2 billion in revenue with a 30% profit margin.^31^ The access and distribution model is therefore of substantial consequence.

There have always been stakeholders and rightsholders, those with an economic investment in the production of scientific content and those entitled to distribute biomedical publications. Before the open access movement, toll access journals were both (Table 5). They took the financial risk and collected fees from readers to produce and distribute published works. Unlike musicians, for example, most scholarly authors readily consent to relinquish copyrights in exchange for publication and its associated prestige. Open access allows the author, who pays an article publishing charge (APC), to retain copyrights and provide their work free to the public in hopes of increasing its impact.^34^

**Table 5.**
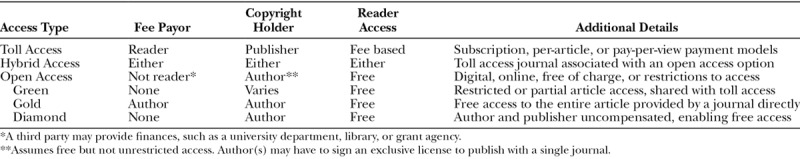
Levels of Access to Publications^32,33^

Before online mass communication, scientific journal open access was not an option. Just as online banking provided an alternative to brick-and-mortar financial institutions,^5^ open access is viable because of low digital publication overhead. Never before has data been shared with so little physical infrastructure, making creative commercial practices possible.^35^ Some forward-thinking editors have embraced open access, epub ahead of print, eprint, and online postpublication review. Rod Rohrich, editor of *Plastic and Reconstructive Surgery*, introduced open access to plastic surgery in North America, calling it “a viable and accepted global distribution model for biomedical publication.”^36^ Its advantages include: faster publication speed, retained author copyrights, wider readership, and free access to publicly funded research. If peer review principles are upheld, most editors laud the open access model’s free dissemination of knowledge. The National Institutes of Health has even made policies to usher in more open access publications.^37^ However, the explosion of the open access journals has also borne witness to specious reviewer ethics, predatory journals, non–peer reviewed pseudoscientific platforms, and poorly reviewed publications. One notable example includes a journal *Ethology* article with a parenthetic quote “should we cite the crappy Gabor paper here?” that survived reportedly multiple levels of peer review and editing opportunities before being published on the article’s first page.^38^ The net effect has either been irreversibly compromised scientific integrity or positive information sharing.

Jeffrey Beall’s online directory of predatory journals illustrates some of the caveats of open access. Beall, a University of Colorado librarian, posted online over 10,000 open access journals that he regarded as sham, profit-seeking, pseudoscientific operations to shame unscrupulous journals and warn potential victims of exorbitant publication fees, or shoddy peer review processes.^39^ This controversial “blacklist” caused an uproar (Table 1). He was subsequently targeted with defamation accusations and took down his blacklist, but raised serious questions about dilettante, self-serving publications, which have subsequently led to greater open access oversight and scrutiny (Table 6).^40^ His example reminds us that the real value of publication lies in legitimate peer review.

**Table 6.**
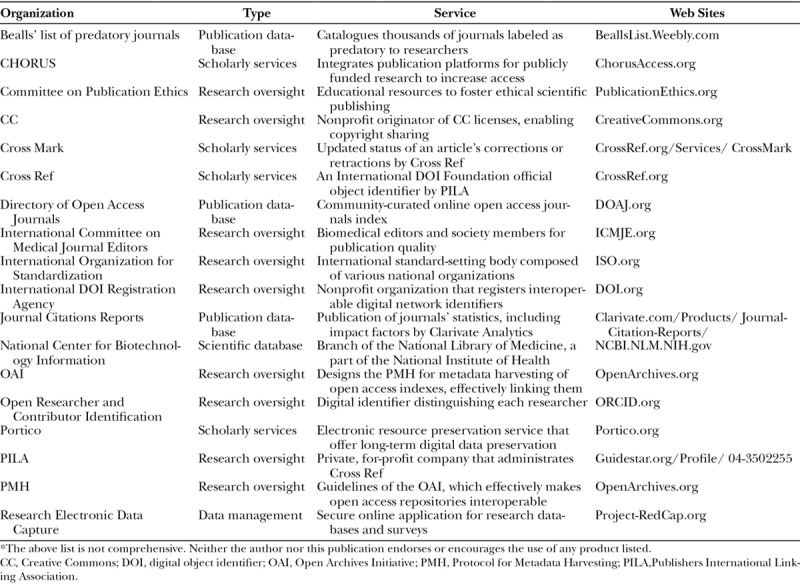
Academic New Media Oversight and Management Organizations

## THE DARK SIDE OF PEER REVIEW

Albert Einstein’s iconic place in history epitomizes genius. He also bristled to peer review. After a critical response from the *Physical Review*, the preeminent physics journal of the day, Dr. Einstein wrote to the editor that he saw “no reason to address the—in any case erroneous—comments of your anonymous expert.”^41^ The paper was later submitted and published elsewhere…interestingly with edits related to the very critiques Dr. Einstein refused to concede.

Peer review is the evaluation of intellectual content by one or more individuals of similar aptitude to the work’s authors. A well-defined, vetted, systematic analysis by qualified experts employing appropriate checks and balances seldom blunders. A rapid expansion of online publications has accompanied the proliferation of new media, sometimes overwhelming the peer review processes. Though plastic surgeons are not responsible for the most humiliating gaffes, notable new media peer review errors betray this strain.

One famous example of sham science was the publication of an article purporting to describe the “midichlorion,” the organelle of “the Force.”^42^ Echoing Luke Skywalker, made-up authors Drs Lucas McGeorge and Annette Kin convinced 7 journals to accept their paper—3 publishing it for free and 4 that requested over $350 each in APCs—despite the inclusion of the following text:

Did you ever hear the tragedy of Darth Plagueis the Wise? I thought not. It is not a story the Jedi would tell you. It was a Sith legend. Darth Plagueis was a Dark Lord of the Sith, so powerful and so wise he could use the Force to influence the midichlorions to create life.^42^

Another ignominious example is a publication by Dr. Ocorrafoo Cobange describing an anticancer chemical isolated from a lichen at the Wassee Institute of Medicine, which turned out to be a complete fabrication; none of these entities exist.^43^ John Bohannon, an investigative journalist, received acceptance from half of the over 300 open access journals; he offered this intentionally fraudulent article, written to expose publishers scamming authors out of up to over $3,000 in APCs. Some journals had false physical addresses within the United States and collected fees via foreign bank accounts. One journal’s editor acknowledged their mistake but demanded payment of the APC anyway.

The above examples’ publication methods were located exclusively online. Such sobering cases illustrate a lack of regulation and accountability within virtual space. It is not that new media is inherently flawed, but the onus is on academicians to exercise vigilance.

## INDICES: THE MORE META THE BETTA

Citation indexing dates to 12th-century Hebrew texts that were the first writings to employ this bibliographic technique.^44^ Until digital media made information-sharing possible with a few lines of code, more sophisticated indexing was not possible. Entire databases can now be generated based on key words, phrases, and even semantic meanings independent of literal syntax.^45^ Another key development was the advent of the Creative Commons license, which allows sharing of copywritten materials (Table 6). These changes have led to ever more expansive open access publication indexes, some employing sophisticated “impact” algorithms, based on number of citations, popular news or social media mentions, or a combination of other altmetric factors (Table 2). This can blur boundaries between publication platform, impact rating, postpublication promotion, and professional networking. All of these components, for example, are featured on the Faculty of 1000 Web portal (Table 7).

**Table 7.**
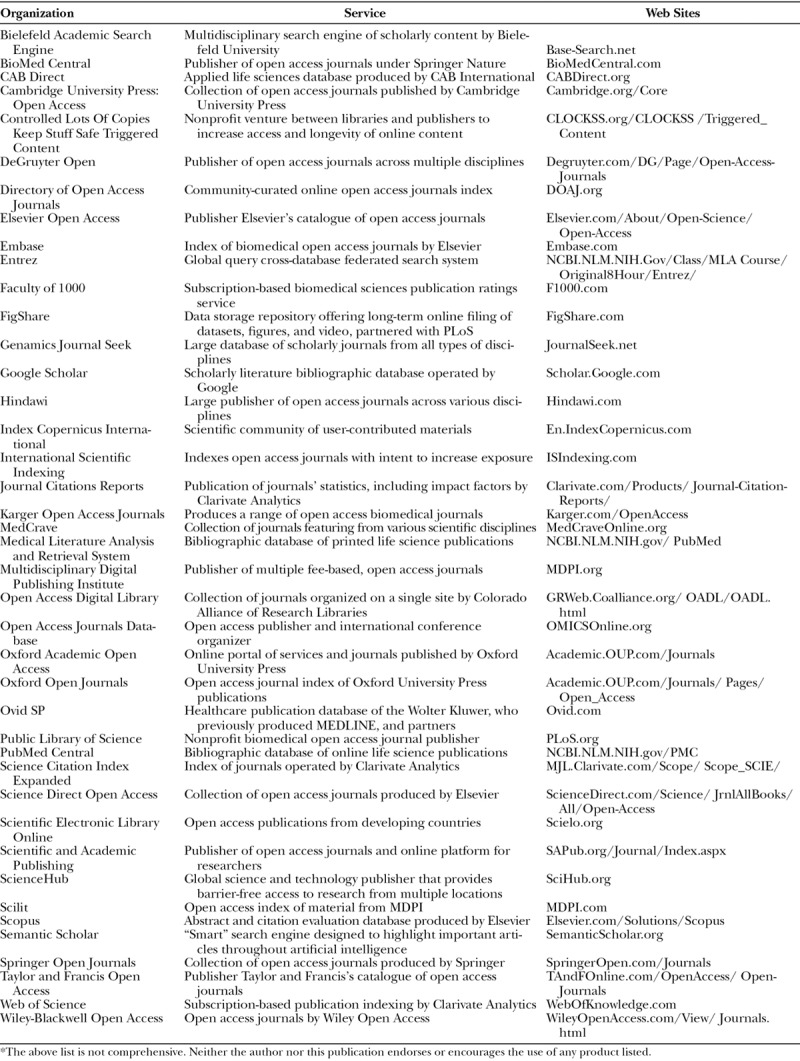
Online Biomedical Journal Databases, Indices, and Search Engines

Various levels of open access journal quality have proliferated, so indexing attempts to make order out of the chaos by consolidating users and subscribers. This has led to an arms race that equates the database size with quality. Multiple “mega-indexes” now boast tens-of-thousands of journal titles and hundreds-of-millions of documents under their auspices. Lower quality and predatory practices, however, have been correlated with increased numbers.^46^ There is the age-old saying about databases that “you get out what you put in,” implying that if the input is of low quality then the product will be as well, regardless of its complexity.

## PREDATOR-IN-CHIEF

Henry Oldenburg was a German theologian, the first secretary of the Royal Society of London, and the founding editor of *Philosophical Transactions*, the world’s first modern scientific journal. He initially published this groundbreaking periodical with personal funds in exchange for the rights to its profits, a venture that paid Oldenburg’s rent in Westminster.^47^ Although scientific publishing is not immune to economic interests, misrepresentation of scholarly intentions for financial gain is an alarming trend within the new media academic industry.^48^

A compelling example of profit-seeking abuse is provided by Katarzyna Pisanski, a researcher in the School of Psychology at the University of Sussex. Dr. Pisanski’s article in the journal *Nature* describes editor applicant Anne O. Szust, who boasted a gamut of credentials created entirely on online on platforms like Academia.edu (Table 4). Despite lacking a single peer-reviewed citation or editorial credentials of any kind, her application was accepted by 48 journals, including 8 from so-called whitelist journals and 4 that made her editor-in-chief.^49^ Her fictitious university affiliation was never vetted, and her cover letter stated that her motive to become editor was to obtain a degree that her fabricated *curriculum vitae* claimed she already had. Dr. Oszust, in fact, never existed. “Oszust” is the Polish word for “fraud.”

More comprehensive journal evaluation tools exist, but 3 questions efficiently discern the merit of scientific journals: (1) Is it indexed on PubMed? (2) What is its impact factor? (3) Is there a publication fee? “Yes,” “>1,” and “no” are reasonable answers, although exceptions certainly exist. Each new media academic participant can quickly develop screening criteria when interacting critically with online organizations.^50^ Dr. Rohrich highlights many red flags in “Top Ways to Spot a Predator”:^51^

1. Payment is required at submission.
2. No reviews are offered and no revisions are requested.
3. All articles are accepted.
4. Does not explicitly follow a standard ethical policy.
5. Sends frequent emails soliciting articles for fast processing.
6. Offers editorial board membership with little criteria.
7. No physical address of phone number to editor or publisher’s office.
8. Grammatical or technical errors on website.
9. No valid International Standard Serial Number.
10. Additional fees requested for steps not previous disclosed (eg, withdrawing, edits, etc.).
11. Not listed on major journal database, such as Scopus, Directory of Open Access Journals, or Web of Science.
12. Solicits articles on topics outside of author’s area of expertise.
13. Difficult opt out of receiving emails after attempts to unsubscribe.
14. If it seems too good to be true, it just might be!

## NAVIGATING ACADEMIC NEW MEDIA

Future new media academic processes promise more integration of cloud-based data, artificially intelligent searches, virtual scientific communities, online quality authorities, altmetrics, open access, mega-indices, and predatory publishing. New media will continue to develop as online platforms’ content, quality, services, and publication ethics evolve. Expect expanded online academia as younger generations advance and older generations learn new media. Whether these changes erode academic purity or not, the age of new media scholarship, or “escholarship,” is upon us. It is essential that plastic surgeons grasp its fundamentals to properly participate in online knowledge sharing. The field of plastic surgery has avoided the embarrassing examples above, but is susceptible to misrepresentation due to its high public profile and lay person appeal. Only through continued vigilance will the field remain unscathed.

Navigating the academic new media landscape requires evidence-based principles, critical thinking, and learning about digital trends. How plastic surgeons negotiate virtual scholarly environments will define the new media digital academic complex moving forward. But Heather Furnas cautions that “ultimately, we should be looking at how to expand, not limit, our audience reach.”^52^ Focusing too heavily on new media dangers may limit the potential for positive impact, but an appreciation for new media’s pitfalls by scholars will help ensure that it is used to benefit patients.
